# Discovery of a biomarker candidate for surgical stratification in high-grade serous ovarian cancer

**DOI:** 10.1038/s41416-020-01252-2

**Published:** 2021-01-21

**Authors:** Haonan Lu, Paula Cunnea, Katherine Nixon, Natasha Rinne, Eric O. Aboagye, Christina Fotopoulou

**Affiliations:** 1 Department of Surgery and Cancer, Division of Cancer, Faculty of Medicine, Imperial College London, London, W12 0HS UK; 2 Department of Surgery and Cancer, Cancer Imaging Centre, Faculty of Medicine, Imperial College London, London, W12 0HS UK

**Keywords:** Prognostic markers, Ovarian cancer

## Abstract

**Background:**

Maximal effort cytoreductive surgery is associated with improved outcomes in advanced high-grade serous ovarian cancer (HGSOC). However, despite complete gross resection (CGR), there is a percentage of patients who will relapse and die early. The aim of this study is to identify potential candidate biomarkers to help personalise surgical radicality.

**Methods:**

136 advanced HGSOC cases who underwent CGR were identified from three public transcriptomic datasets. Candidate prognostic biomarkers were discovered in this cohort by Cox regression analysis, and further validated by targeted RNA-sequencing in HGSOC cases from Imperial College Healthcare NHS Trust (*n* = 59), and a public dataset. Gene set enrichment analysis was performed to understand the biological significance of the candidate biomarker.

**Results:**

We identified *ALG5* as a prognostic biomarker for early tumour progression in advanced HGSOC despite CGR (HR = 2.42, 95% CI (1.57–3.75), *p* < 0.0001). The prognostic value of this new candidate biomarker was additionally confirmed in two independent datasets (HR = 1.60, 95% CI (1.03–2.49), *p* = 0.0368; HR = 3.08, 95% CI (1.07–8.81), *p* = 0.0365). Mechanistically, the oxidative phosphorylation was demonstrated as a potential biological pathway of *ALG5*-high expression in patients with early relapse (*p* < 0.001).

**Conclusion:**

*ALG5* has been identified as an independent prognostic biomarker for poor prognosis in advanced HGSOC patients despite CGR. This sets a promising platform for biomarker combinations and further validations towards future personalised surgical care.

## Background

The clinical implications of adverse tumour biology and their direct effects on clinical decision-making processes have not yet been fully elucidated or defined in patients with high-grade serous ovarian cancer (HGSOC). This represents a major challenge compared to other neoplasms, such as colon or breast cancer, where defined signatures of tumour biology are becoming integrated into the clinical management and treatment pathways of affected patients.^1,2^

The cornerstone of treatment for advanced HGSOC has long been a combination of maximal effort cytoreductive surgery paired with platinum-based chemotherapy.^3^ Recently, the concept of a maintenance approach targeting processes, such as DNA damage response and angiogenesis has been implemented in an effort to increase remission rates and optimise outcomes, especially for those patients with BRCA mutations or high risk disease.^4,5^ Although, there has been some progress in allocating HGSOC patients to the appropriate maintenance treatment according to their genomic (germline or somatic) mutational profiles,^4,5^ to date, no biological stratification models exist for surgical cytoreduction. Currently, all surgical decision-making processes are based mainly on infrastructural, clinical and fragility parameters as well as factors related to surgical philosophy and training.^6^

It is well accepted that incorporating maximal effort or radical surgery into the initial management of HGSOC significantly contributes to more favourable outcomes for patients, including those with high tumour burden and disseminated disease.^7^ Nevertheless, prospective and retrospective data indicate that there is a cohort of patients who will experience early relapse and have less favourable outcome despite complete gross resection (CGR).^8,9^ While there have been many efforts in identifying the patient subgroup that will end up with visible post-operative residual disease,^10^ studies focusing on identifying those patients that will have an unfavourable outcome despite CGR are scarce and findings are often influenced by suboptimal or inhomogeneous surgical quality.^11,12^

The present work attempts to address this unmet need and describes the identification and validation of a transcriptomic biomarker, using multiple HGSOC patient cohorts, that could reliably predict those patients that may not benefit from extensive, multi-visceral cytoreductive techniques, and should therefore have a more tailored approach. Consequently, in the future, we could help personalise surgical treatment for all patients and direct radicality towards those that will benefit most and avoid any unnecessary surgical morbidity in patients without real survival benefit.

## Methods

### Public datasets

Four Affymetrix U133 publicly available datasets from patients with high-grade serous ovarian cancer (HGSOC) were downloaded and used in this study: The Cancer Genome Atlas (TCGA),^13^ the Tothill (GSE9891) patient cohort,^14^ the Charité University Hospital, Berlin, patient cohort (GSE14764)^15^ and the UVA-55 cohort (GSE30161).^16^ The raw CEL data were downloaded from Gene Expression Omnibus (https://www.ncbi.nlm.nih.gov/geo/) with accession numbers GSE82191 (TCGA), GSE9891 (Tothill), GSE14764 (Charité) and GSE30161 (UVA-55), respectively. The combined cohorts of TCGA, Tothill and Charité are referred to the TTC cohort and were used as the training dataset for analysis. The UVA-55 cohort was used as a second independent validation dataset. The clinical characteristics of patients in TTC, HH and UVA-55 cohort are summarised in Table 1 and Supplementary Table 1. CGR refers to patients with complete gross resection of tumour burden, non-CGR refers to patients with any remaining residual disease.

**Table 1:** Patient characteristics in the TTC and HH cohorts.

Characteristics	TTC (*n* = 536)	HH (*n* = 126)	*p*-value
Age (%)			
≤60 >60 Unknown	270 (50.4) 208 (38.8) 58 (10.8)	65 (51.6) 61 (48.4) –	ns
Stage (%)			
III IV	479 (89.4) 57 (10.6)	93 (73.8) 33 (26.2)	<0.0001
Post-operative residual disease (%)			
CGR Non-CGR	136 (25.4) 400 (74.6)	82 (65.1) 44 (34.9)	<0.0001
Follow-up (FU; months)			
Median IQR	46.2 25.0–75.3	46.3 17.7–67.9	ns
PFS (months)			
Median IQR	16.0 10.8–28.2	18.4 11.7–37.3	<0.0001
PFS (months)			
CGR Non-CGR	21.1 15	23.4 13.3	ns ns
OS (months)			
Median CI	41.0 37.3–45.3	58.2 50.1–65.9	0.0017
Relapsed within the FU time (%)			
No Yes	169 (31.5) 367 (68.5)	53 (42.1) 73 (57.9)	0.0317

### Patient summary for in-house dataset

An in-house cohort of patients with advanced HGSOC treated in the Hammersmith Hospital, Imperial College Healthcare NHS Trust was accessed to use as an additional independent validation cohort. The sample size was estimated based on the Cox regression analysis from the TTC cohort (training dataset) and using power of 80% and alpha of 0.05, 54 CGR cases were calculated to be required for validation by RNA-sequencing. Tumour samples (CGR: *n* = 59; non-CGR: *n* = 26) and anonymised clinical data (*n* = 126) from patients with advanced HGSOC treated between 2005 and 2015 were retrospectively collected and accessed under the Hammersmith and Queen Charlotte’s & Chelsea Research Ethics Committee approval 05/QO406/178. The progression-free survival was determined as the time interval between the end of the event of interest (i.e. surgery) till the first defined event of relapse for this patient. CA125 increase alone without clinical or radiological correlation is not being considered as relapse event. According to the GCIG criteria, response to chemotherapy is being defined on biochemical (i.e. CA125 levels) as well as radiological (i.e. CT /MRI) and clinical level (i.e. ascites, pleura effusion, performance status, etc.).^17^ Patients who progressed or relapsed during or within 6 months after platinum-based chemotherapy were classified as platinum refractory/ resistant and those who did not experience a relapse during follow-up or did so at a longer time interval than 6 months after last platinum treatment were defined as platinum sensitive. To note, both the Charité University Hospital and Hammersmith Hospital Imperial College Healthcare NHS centres are certified centres of excellence for ovarian cancer surgery by the European Society of Gynaecologic Oncology (ESGO).^18^ The inclusion criteria of the patients in this study were: (1) Advanced (FIGO stage III–IV^19^) HGSOC patients who have undergone primary cytoreductive surgery followed by platinum-based chemotherapy, (2) available tumour samples snap frozen and banked following primary surgery. The exclusion criteria were: (1) Patients with relapsed disease or previous treatment for other cancers; (2) banked samples of the patient with poor tumour tissue quality (e.g. low tumour cellularity and RNA quality). Histological review of tumour samples collected was performed by expert gynae-pathologists based in the West London Gynae Cancer Centre at Hammersmith hospital, Imperial College Healthcare NHS Trust. This study followed the REMARK criteria for prognostic biomarker discovery.^20^ Table 1 lists the clinico-pathological data of the data and samples used.

### Targeted RNA sequencing

RNA was extracted from fresh frozen tumour tissues (*n* = 85) from the Hammersmith Hospital cohort (HH cohort), including only patients that had primary cytoreductive surgery and CGR, using the protocol described elsewhere in detail.^12^ Briefly, each frozen tumour was lysed using a Tissuelyser II (QIAGEN, UK) at 15 Hz for 2 min. RNA was then extracted using the RNeasy kit (QIAGEN, UK) following the manufacturer’s protocols and quantified using the Bioanalyzer system (Agilent, USA).

To quantify the mRNA expression of *ALG5*, *GPR107* and *NUP188* in each tumour sample, total RNA from each individual case was firstly reverse transcribed into cDNA using ProtoScript® II Reverse Transcriptase (NEB, USA). The cDNAs were then amplified with a pool of indexed primers using TruSeq Targeted RNA Custom Panel Kit (Illumina, USA). The primers were selected using the Illumina DesignStudios. The amplicon sizes in the cleaned PCR product were quality controlled using a TapeStation (Agilent, USA). Next, forty-eight samples were multiplexed in one single MiSeq run, with SR 50 setting which generated 20 million reads per run. The experimental procedures were performed blinded from the study end-point.

### TTC and UVA-55 cohort data normalisation

Independent gene expression microarray studies often show significant batch effect. To minimise any batch effect within the combined TTC cohort which originated from three independent studies, frozen robust multi-array analysis (fRMA) methodology was first applied to normalise the three microarray datasets (TCGA, Tothill and Charité). The fRMA method was chosen as it is particularly robust when combining multiple microarray datasets for analysis. Briefly, fRMA function from ‘frma’ package was applied with arguments of background = ‘none’, normalise = ‘quantile’, summarise = ‘robust_weighted_average’ and target = ‘probeset’. Next, the three fRMA normalised datasets were combined and batch-adjusted by COMBAT using ‘merge’ function from ‘inSilicoMerging’ package in R.

The UVA-55 dataset (GSE30161) was normalised using the robust multi-array average method from ‘affy’ package in R.

### Probe selection for biomarker discovery analysis

Affymetrix microarray platforms contain multiple probes for individual genes, however, filtering should be performed to remove low-quality probes. To solve this issue, we correlated microarray probes with RNA-sequencing (RNAseq) data using gene names from the TCGA ovarian cancer dataset. Microarray probe: RNAseq pairs with a Pearson coefficient <0.6 were removed. Furthermore, among the multiple probes that target a single gene, the probe that showed the strongest positive Pearson correlation with the corresponding RNAseq data was retained for downstream analysis. As a result, 9207 probes corresponding to unique genes were included in the downstream biomarker discovery analysis.

### Survival analysis

To identify potential significant biomarkers of surgical outcome in the TTC cohort, all 9207 probes were correlated with progression-free survival (PFS) as the primary study end-point using Cox proportional hazard regression. The *p*-values generated were adjusted for multiple testing using the Benjamini & Hochberg procedure. The candidate biomarkers were then tested for their independence by adjusting the Cox model for FIGO stage and age. The C-index as a measure of survival model fitting was calculated using ‘coxph’ function in ‘survival’ package. All the Kaplan–Meier analyses in this study were performed using ‘ggkm’ function from ‘ggkm’ package in R. The cut-off for *ALG5*-high and *ALG5*-low was defined by *ALG5* expression at upper tertile in TTC, HH and UVA-55 cohorts.

### Gene set enrichment analysis

To discover potential biological pathways associated with *ALG5* expression, each gene in the TCGA ovarian cancer gene expression microarray dataset was correlated with *ALG5* using ‘cor.test’ function in R 3.2.2. The global genes were ranked by the *p*-values in negative log10 ratio. The ranked gene list (Supplementary Table 2) was then used in the GSEA software (http://software.broadinstitute.org/gsea/index.jsp; Broad Institute), with 1000 permutations, classic enrichment statistic and KEGG as pathway database.

## Results

### Clinical significance of post-operative residual disease in HGSOC

Clinical outcome data from three independent datasets, the Cancer Genome Atlas (TCGA),^13^ the Tothill (GSE9891)^14^ patient cohort and patients from the Charité University Hospital in Berlin (GSE14764)^15^ were combined (*n* = 536; TTC cohort) to assess the impact, prognostic and predictive factors of post-operative residual disease in patients with HGSOC. In addition, an independent cohort, the Hammersmith Hospital patient cohort (*n* = 126; referred as HH cohort below), was used to validate the findings. The patient demographics are summarised in Table 1.

As per the inclusion criteria of the study, all patients within the TTC and HH cohorts underwent primary cytoreductive surgery, followed by platinum-based chemotherapy due to HGSOC. The majority of the patients: 479 (89.4%) in the TTC and 93 (73.8%) in the HH cohorts were FIGO stage IIIa-c; while 136 (25.4%) and 82 (65.1%) patients, respectively, underwent a CGR. Median follow-up time was 46.2 months (IQR: 25.0–75.3; TTC) and 46.3 months (IQR: 17.7–67.9; HH).

**Fig. 1:**
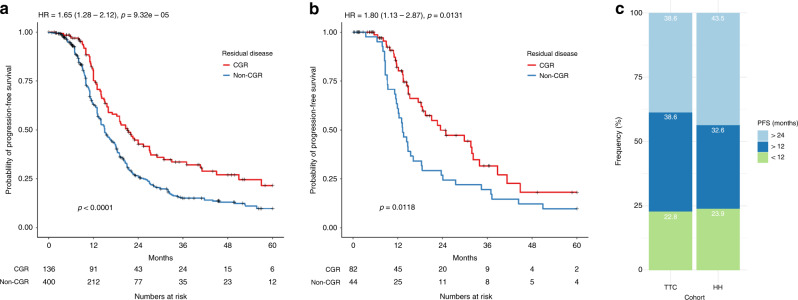
Complete gross tumour resection is associated with improved prognosis in advanced stage high-grade serous ovarian cancer patients. Kaplan–Meier analysis associating post-operative residual disease status with progression-free survival in **a** the combined TCGA, Tothill and Charité cohort (TTC) and **b** Hammersmith cohort (HH). Only HGSOC patients with FIGO stage III–IV are included. *P*-values from log-rank test are indicated in the bottom left. Hazard ratios (HR) and *p*-values from Cox proportional hazard regression are indicated on the top left. **c** Proportion of patients with PFS of <12 months, >12 months and <24 months, and >24 months in the TTC and HH cohort. HGSOC patients with post-operative CGR status and FIGO stage III–IV are included in this analysis.

In the TTC cohort, advanced stage HGSOC patients (FIGO stage III–IV) that had undergone CGR had a median progression-free survival (PFS) of 21.1 months compared to 15 months in patients with residual disease (HR: 1.65, 95% Confidence Interval (CI) 1.28–2.12, *p* < 0.0001); Fig. 1a. Equally, in the HH cohort, patients with CGR had a median PFS of 23.4 months compared to 13.3 months in patients with post-operative residual disease (HR: 1.80, 95% CI 1.13–2.87, *p* = 0.0131); Fig. 1b. Of those patients with CGR, 22.8% in the TTC-cohort and 23.9% in the HH-cohort, relapsed within one year following primary cytoreductive surgery, while an additional 38.6% (TTC) and 32.6% (HH) patients relapsed within two years after primary cytoreductive surgery (Fig. 1c). The clinical characteristics of the patients with early relapse was summarised in Supplementary Table 3.

We tested the prognostic potential of two existing prognostic markers, molecular subtypes (differentiated, immunoreactive, mesenchymal and proliferative) and FIGO stage, in the TCGA and Tothill datasets, and combined TTC and HH CGR cohorts respectively (Supplementary Fig. 1a–e). However, no statistically significant associations were observed between the two markers and progression-free survival, highlighting the need for discovery of novel biomarkers.

### Discovery and validation of a prognostic biomarker for surgical outcome

**Fig. 2:**
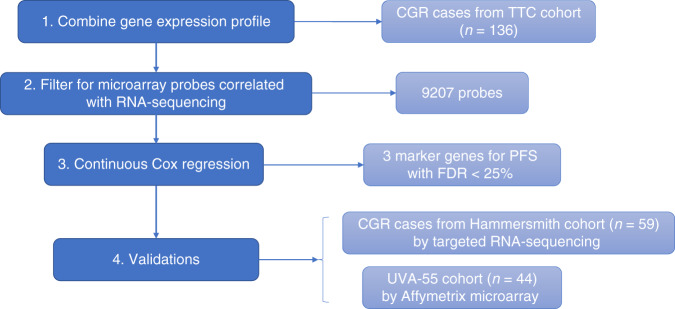
Biomarker discovery workflow. 1 HGSOC patients with FIGO stage III–IV and CGR from TCGA, Tothill and Charité datasets were identified; Affymetrix U133 microarray-based gene expression profile from the TTC cohort were combined and adjusted for batch effect; 2 Unique probes from Affymetrix U133 microarray that had >0.6 Pearson coefficient with corresponding RNA-sequencing data in the TCGA dataset were identified and selected. 3 Continuous Cox proportional hazard regression model was applied for the 9207 probes. Three candidate biomarker genes for PFS with FDR < 25% were identified. 4 The three candidate biomarkers were validated in the HH cohort by targeted RNA-sequencing, and then validated in the UVA-55 (GSE30161) cohort by Affymetrix U133 microarray.

The gene expression microarray datasets for patients with CGR in the TTC cohort were combined (Supplementary Fig. 2) and used as the training analysis dataset (*n* = 136), to identify prognostic biomarkers for patients with less favourable prognosis despite having undergone a CGR (Fig. 2).

In the TTC cohort, three genes, *ALG5*, *GPR107* and *NUP188*, were significantly associated with PFS in the Cox proportional hazards regression analysis, using the mRNA expression level of each gene as a continuous variable. The three genes had hazard ratios (HRs) of 1.76 (95% CI: 1.35–2.30; *p* < 0.0001; range: 7.73–11.3; C-index: 0.623), 0.591 (95% CI: 0.456–0.765; *p* < 0.0001) and 0.647 (95% CI: 0.522–0.803; *p* < 0.0001), respectively; and these associations were below a false discovery rate (FDR) of 25%. The expression of *ALG5*, *GPR107* and *NUP188* appear to be strongly correlated with each other: *ALG5* is inversely correlated with *GPR107* (Pearson *r* = −0.168, *p* = 0.0511) as well as *NUP188* (Pearson *r* = −0.402, *p* < 0.0001); whereas *GPR107* and *NUP188* are positively correlated with each other (Pearson *r* = 0.306, *p* < 0.001; Supplementary Fig. 3) in the TTC cohort.

In addition, a targeted RNA-sequencing experiment was performed using tumour samples collected from patients who had undergone a CGR at upfront cytoreductive surgery within the HH cohort as an independent validation set (*n* = 59). The candidate biomarkers were further validated using an additional independent gene expression microarray dataset from the UVA-55 cohort (*n* = 44; Supplementary Table 1).^16^

Among the three genes, only *ALG5* retained its association with the PFS in the multivariable Cox proportional hazards regression model in the HH cohort (HR = 1.62, 95% CI: 1.06–2.49, *p* = 0.0255; C-index: 0.6; Table 2), and UVA-55 cohort (HR = 3.36, 95% CI: 1.05–10.7, *p* = 0.0406; C-index: 0.692, Table 2). These associations were independent of known clinical prognostic factors, including age and FIGO stage (Supplementary Fig. 4a, b and Supplementary Table 4). In addition, *ALG5* expression remains consistent across primary ovarian tumour, omental tissue and peritoneal diseases (Supplementary Fig. 4c). *GPR107* and *NUP188* failed to retain any prognostic significance for PFS in the HH or UVA-55 validation cohorts.

**Table 2:** Summary of Cox proportional hazard regression of ALG5, GPR107, NUP188 in TTC, HH and UVA-55 cohorts.

		Univariate			Multivariable^a^		
Cohort	Gene symbol	Hazard ratio	95% CI	*p*-value	Hazard ratio	95% CI	*p*-value
TTC	ALG5 (218203_at)	2.42	1.57–3.75	*p* < **0.0001**	1.90	1.10–3.29	**0.0209**
	GPR107 (211979_at)	0.337	0.199–0.574	*p* < **0.0001**	0.361	0.190–0.686	**0.00185**
	NUP188 (212691_at)	0.340	0.199–0.581	*p* < **0.0001**	0.260	0.126–0.536	**0.000263**
HH	ALG5	1.60	1.03–2.49	**0.0368**	1.62	1.06–2.49	**0.0255**
	GPR107	0.948	0.343–2.63	0.92	0.801	0.273–2.36	0.687
	NUP188	0.984	0.671–1.44	0.935	0.953	0.647–1.41	0.810
UVA-55	ALG5 (218203_at)	3.08	1.07–8.81	**0.0365**	3.36	1.05–10.7	**0.0406**
	GPR107 (211979_at)	0.451	0.117–1.75	0.249	0.447	0.113–1.77	0.252
	NUP188 (212691_at)	0.398	0.11–1.44	0.160	0.289	0.0616–1.36	0.116

Bold values indicate statistical significance *p* < 0.05.

^a^Multivariable analysis adjusted for stage and age. Gene expression, stage and age as continuous variables.

**Fig. 3:**
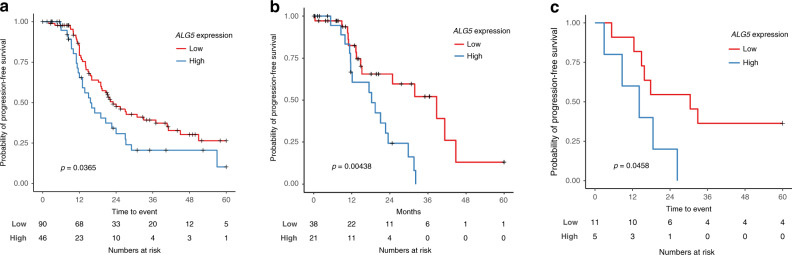
The prognostic associations of *ALG5* expression in CGR-operated HGSOC patients. Kaplan–Meier analysis associating *ALG5* mRNA expression with progression-free survival in the **a** TTC training set cohort and in two independent validation cohorts from **b** HH and **c** UVA-55. HGSOC patients with FIGO stage III–IV and CGR are included in the analysis. *P*-values from log-rank test are indicated.

In addition to the Cox regression analysis which used *ALG5* expression level as a continuous variable, Kaplan–Meier analysis was also performed to confirm the association of dichotomised *ALG5* expression with PFS in each cohort (Fig. 3). The patients with CGR in the *ALG5*-high subgroup had a median PFS of 15.6 months (TTC), 18.3 months (HH) and 14.5 months (UVA-55) compared to 27.3 months (TTC), 38.8 months (HH) and 32.9 months (UVA-55) in the *ALG5*-low CGR subgroup.

We further examined the CGR and non-CGR patient subgroups in both HH and UVA-55 cohorts (Supplementary Fig. 5). As expected, CGR HGSOC patients in the *ALG5*-low subgroup showed a trend of better PFS compared to non-CGR patients (HH: HR = 0.543, 95% CI: 0.271–1.09, *p* = 0.0838; UVA-55: HR = 0.3, 95% CI: 0.0858–1.05, *p* = 0.0593). However, the association between surgical outcome and PFS diminished in the *ALG5*-high subgroup (HH: HR = 0.32, 95% CI: 0.0657–1.56, *p* = 0.159; UVA-55: HR = 0.796, 95% CI: 0.335–1.89, *p* = 0.604), indicating a potential stronger value of *ALG5*-high expression over the presence of post-operative residual disease.

Previous studies have reported the promoter DNA methylation status of *MYLK3*^11^ and a CT-image based radiomics prognostic vector (RPV) signature^12^ as potential predictive biomarkers for clinical and surgical outcome in HGSOC patients. We, therefore, tested if there was an association of *ALG5* expression and these proposed biomarkers (Supplementary Fig. 6). No relevant correlations were observed, between *ALG5* and *MYLK3* methylation status or between *ALG5* expression and RPV. Moreover, *ALG5* remained as an independent prognostic factor even after inclusion of the *MYLK3* methylation and RPV in the multivariable Cox regression model (Supplementary Fig. 6). Furthermore, *ALG5* expression was not significantly associated with the established general prognostic molecular subtypes biomarkers in HGSOC (Supplementary Fig. 7).

### *ALG5* expression is associated with oxidative phosphorylation

To better understand the potential biological significance of the variability of *ALG5* expression, we identified the biological pathways correlated with *ALG5* expression, using pre-ranked gene set enrichment analysis (GSEA).^21^

**Fig. 4:**
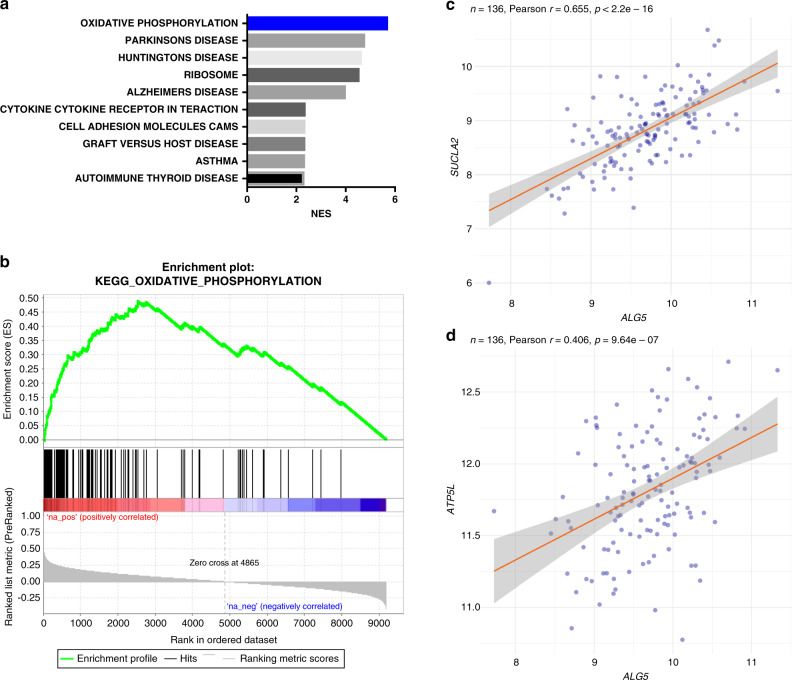
*ALG5* correlates with oxidative phosphorylation pathway. **a** Biological pathways correlated with *ALG5* expression from gene set enrichment analysis. **b** Enrichment plot of oxidative phosphorylation pathway associated with *ALG5* expression. Scatter plot showing *ALG5* correlation with **c***SUCLA2* and **d***ATP5L* using the TTC cohort. Pearson correlation coefficients and *p*-values are indicated.

**Fig. 5:**
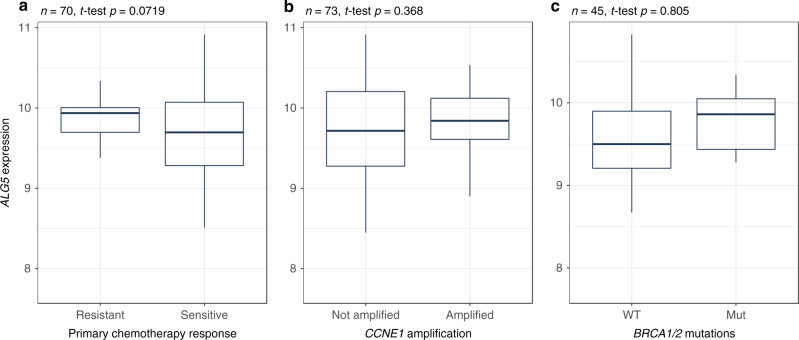
*ALG5* expression may not directly predict primary chemotherapy response. The associations between *ALG5* mRNA expression and **a** primary chemotherapy response, **b***CCNE1* amplification and **c***BRCA1/2* mutation status in the TCGA dataset. *P*-values from two-tailed *t*-test are indicated.

The list of pathways that are significantly associated with *ALG5* expression from the Kyoto Encyclopaedia of Genes and Genomes (KEGG) database is shown in Fig. 4a. The oxidative phosphorylation pathway ranks as the most significantly associated pathway (*p* < 0.001, FDR < 1%) and is also closely linked to the known function of ALG5 (Dolichyl-phosphate beta-glucosyltransferase), which facilitates glucose transfer during N-linked protein glycosylation.^22,23^ The enrichment plot illustrates the enrichment of oxidative phosphorylation-related genes in the *ALG5*-correlated gene list: 75.8% of the genes in the oxidative phosphorylation pathway are among the top *ALG5*-correlated genes (leading edge; Fig. 5b and Supplementary Table 2). As a validation, correlations between *ALG5* and two known genes in the oxidative phosphorylation pathway, *SUCLA2* or *ATP5L*, are shown (Fig. 4c, d), again confirming the strong correlation between *ALG5* and the oxidative phosphorylation pathway.

### *ALG5* expression is independent of response to platinum-based chemotherapy

To delineate whether the prognostic value of *ALG5* is also potentially associated with chemotherapy response due to a known link between oxidative phosphorylation and platinum sensitivity,^24–26^ we investigated whether there was any correlation between *ALG5* expression and chemotherapy response in the TCGA and HH datasets (Fig. 5a and Supplementary Fig. 8). We could not identify any significant association between *ALG5* and chemotherapy response.

Furthermore, we tested whether *ALG5* is associated with any known genetic factors that may contribute to chemotherapy response, including *CCNE1* amplification and *BRCA1/2* mutations. *ALG5* expression was not associated with any of these predictive biomarkers, highlighting its independent prognostic value (Fig. 5b, c).

## Discussion

In the present study, we have identified and independently validated a prognostic biomarker that could help predict progression of HGSOC in patients who have undergone complete gross resection followed by adjuvant chemotherapy. The prognostic value of this novel biomarker, *ALG5*, appears to be independent from other well-known prognostic factors such as age, FIGO stage and molecular subtypes. By combining with existing prognostic markers, *ALG5* could be used as part of clinical pathway to guide treatment options. Hence, *ALG5* expression may indicate a distinct biological mechanism reflective of post-operative remission, independent of therapeutic effort.

Cytoreductive surgery for advanced ovarian cancer has experienced a vast evolution over the last decades, reaching from an initial nihilism towards a presumed ‘hopeless’ disease, to the development of ‘ultraradical’ debulking techniques in an effort to reach the ‘holy grail’ of complete tumour resection. However, this increased radicality has not come without a cost. Increased surgical complications, longer theatre times and hospital stays, exhaustion of infrastructural and financial resources have continued to present a challenge, especially for healthcare systems with limited availability and support.^27^ Most researchers have focused on identifying those patients who cannot have complete gross resection, despite maximal surgical effort, specialised training skills and optimal infrastructural support. However, so far there has been a failure to identify, establish and validate prognostic and predictive biomarkers that could reliably stratify patients to the appropriate surgical pathway towards personalised surgical care. The ultimate goal would be to direct surgical radicality towards those who will benefit most, sparing those patients, who will not gain any benefit, from unnecessary surgical morbidity. We demonstrated across multiple cohorts of HGSOC patients with CGR, that patients with low *ALG5* expression have a significantly longer PFS than those with high *ALG5*. Importantly, we have also shown that in patients with low *ALG5* expression, surgical effort does indeed matter, in that those patients with CGR have significantly better survival than patients with residual disease of any type. This finding contradicts current arguments towards the value of surgery in advanced ovarian cancer, which claim that it is mainly the tumour biology that dictates survival and also operability and not actual surgical effort.^6,27^ Our data indicate that tumour debulking is an important factor for patient survival and maximal surgical effort can shift the patients towards a more favourable prognosis group.

*ALG5* expression is not associated with known prognostic biomarkers for ovarian cancer including molecular subtypes, *MYLK3* promoter methylation or CT-based biomarker RPV, suggesting *ALG5* may represent a novel biological pathway not affected by CGR. Molecular subtypes were originally derived from transcriptomic data, and the mesenchymal or C1 subtype was shown associated with poor prognosis in HGSOC.^14^ Nevertheless, we did not observe a significant association between molecular subtypes and PFS in the CGR cohorts. Moreover, the prognostic value of *ALG5* was also independent of any interaction or influence on platinum response.

In addition, we demonstrated that *ALG5* expression was significantly associated with a number of biological pathways, including oxidative phosphorylation (*p* < 0.001), which further indicates a prognostic value for *ALG5*. Oxidative phosphorylation, or OXPHOS, is a metabolic process, which generates ATP by transporting electrons across a series of mitochondrial transmembrane protein complexes.^28^ Many cancer types were found to prefer glycolysis instead of OXPHOS to produce ATP, so called ‘Warburg effect’.^28^ Nevertheless, it has been observed that a subset of cancers have an intact mitochondrial metabolism.^29^ Based on this finding, a number of small molecule inhibitors including metformin, arsenic trioxide and CAI (Carboxyamidotriazole) have been used or developed to target the OXPHOS pathway.^29,30^ Studies have also shown that ovarian cancer cells and ovarian cancer stem cells display heterogeneity in their selection of an energy source,^31–33^ and suggest that this flexibility in using either glycolysis or OXPHOS may signal a survival adaptation.^31^ It would therefore be interesting to test whether *ALG5*-high tumours are sensitive to inhibitors of the OXPHOS pathway, as an alternative therapeutic strategy for patients with CGR at early relapse.

A limitation of the present study is the inhomogeneity in surgical techniques and approach, as reflected on the highly disparate total CGR rates between the different cohorts analysed, that may dilute the implementation of our findings in a purely maximal effort surgical setting. Also, further analysis is required to investigate whether the identified biomarker could be also predictive of overall survival.

As a next step, we propose the prospective validation of *ALG5* expression in a multi-centre cohort of patients undergoing maximal effort cytoreductive surgery in specialised centres for ovarian cancer surgery with established surgical quality as per objective criteria.^18^ Furthermore, the combination of gene expression and methylation profiling, and imaging-based approaches may lead to the establishment of a more robust prognostic model.

In summary, we have identified a novel prognostic biomarker that may identify HGSOC patients with a more adverse tumour biology, that are less likely to benefit from radical surgical cytoreduction. Prospective validation studies and even a future prospective randomised controlled trial allowing stratification of patients according to their tumour biology profiles are warranted before the safe implementation of any new biomarker into the clinical routine. In the future, this potential biomarker, together with other existing markers or factors, could be combined to generate a score for surgical benefit, allowing stratification of patients to personalised surgical options and alternative treatment pathways.

## Supplementary information

Supplementary Figures and Tables

Supplementary Table 2

## Data Availability

The RNA-sequencing data and clinical annotations are accessible in Mendeley Data with the identifier 10.17632/kw736vjpsd.1.
