# Supplementary material for: Discovery of a biomarker candidate for surgical stratification in high-grade serous ovarian cancer
Source: Br J Cancer. 2021 Jan 21;124(7):1286–93. doi: 10.1038/s41416-020-01252-2 (PMC8007618; doi:10.1038/s41416-020-01252-2)
Supplement: Supplementary file 1 — Supplementary Figures and Tables [file 41416_2020_1252_MOESM1_ESM.docx]

**Supplementary Figure 1. Association between existing prognostic markers and progression-free survival in CGR cohorts.** Kaplan-Meier plots showing association between molecular subtypes and PFS in **a), c)** CGR cohorts and **b), d)** non-CGR cohorts. The two sets of cohorts are from **a), b)** Tothill dataset and **c), d)** TCGA dataset. Kaplan-Meier analyses showing associations between FIGO stage and PFS in **e)** HH cohort and **f)** TTC cohort. P-values are computed from log-rank test and shown in the plots. DIF, differentiated; IMR, immunoreactive; MES, mesenchymal and PRO, proliferative.

**Supplementary Figure 2. Comparison of gene expression data in TTC cohort before and after normalisation.** Boxplot of gene expression ranges for each individual case and all cases in the TCGA, Tothill and Charité cohorts **a), b)** before and **d), e)** after fRMA normalisation and COMBAT adjustment. Multidimensional scaling plot of combined gene expression data in the TTC cohort **c)** before and **f)** after fRMA normalisation and COMBAT adjustment.

**Supplementary Figure 3. *ALG5* expression is correlated with *NUP188* and *GPR107*.** Scatter plot of *ALG5* and **a** *NUP188* or **b** *GPR107* in TTC cohort and **c** Scatter plot of *NUP188* and *GPR107* in TTC cohort. Pearson correlation coefficients and p-values are indicated.

**Supplementary Figure 4. Association between *ALG5* with known clinical factors. a)** Scatter plot of age by *ALG5* expression in the TTC cohort. Pearson correlation coefficient is given. **b)** Boxplot of *ALG5* expression by FIGO stage in the TTC cohort, p-value is given by two-tailed t-test. **c)** Boxplot of ALG5 expression by disease sites in the Tothill dataset. P-value is given by Kruskal-Wallis test.

**Supplementary Figure 5. Prognostic association of residual disease in *ALG5* expression-stratified subgroups in a) HH and b) UVA-55 cohort.** P-value is given by log rank test.

**Supplementary Figure 6. The prognostic value of *ALG5* is independent of *MYLK3* methylation and RPV.** Scatter plot correlating *ALG5* mRNA expression with **a)** *MYLK3* methylation in the TCGA dataset and **b)** RPV in the HH dataset. **c)** Cox proportional hazard regression of *ALG5* expression and progression free survival with *MYLK3* methylation and RPV as a covariate in the multivariable analysis.

**Supplementary Figure 7. *ALG5* expression is not associated with molecular subtypes defined by a) the TCGA study or b) the Tothill study.** P-values were given by Kruskal-Wallis test. DIF, differentiated; IMR, immunoreactive; MES, mesenchymal and PRO, proliferative.

**Supplementary Figure 8. Association between *ALG5* expression with primary chemotherapy response in HH cohort.** P-value is given by 2-sided t-test.

Supplementary Table 1. Patient characteristics in the UVA-55 cohort.

| **Characteristics** | | **UVA-55 (n= 44)** |
| --- | --- | --- |
| Age (%) | ≤60  >60  Unknown | 20 (45.5)  24 (43.6)  - |
| Stage (%) | III  IV | 40 (90.9)  4 (9.1) |
| Residual disease (%) | CGR  Non-CGR  Unknown | 18 (40.9)  25 (56.8)  1 (2.3) |
| Follow-up (FU; months) | Median  IQR | 84.7  70.3-128 |
| PFS (months) | Median  IQR | 13.2  8.69-19.0 |
| PFS (months) | CGR  Non-CGR | 18.6 (14.1-84.7)  11.2 (8.03-16.6) |
| OS (months) | Median  CI | 37.8  19.5-91.6 |
| Relapsed within the FU time (%) | No  Yes  Unknown | 2 (4.5)  39 (88.6)  3 (6.8) |

Supplementary Table 2. Gene list ranked by correlation with *ALG5*.

Supplementary Table 3. Clinical characteristics of CGR patients with early relapse in combined TTC and HH cohorts.

| **Characteristics** | | **Early/intermediate**  **n=65** | **Late**  **n=33** | **p-value** |
| --- | --- | --- | --- | --- |
| Age (%) | ≤60  >60 | 38 (58.5)  27 (41.5) | 18 (54.5)  15 (45.5) | ns |
| Stage (%) | III  IV | 52 (80)  13 (20) | 28 (84.8)  5 (15.2) | ns |
| Follow-up (months) | Median  IQR | 58.4  34.5-60.0 | 60.0  37.2-60.0 | ns |
| PFS (months) | Median  IQR | 13.3  10.7-18.3 | 40.7  30.9- | <0.0001 |
| OS (months) | Median  CI | 44.4  36.0-58 | -  65.5- | <0.0001 |
| Early: PFS<12months; Intermediate: 12months<PFS<24months; Late: PFS>24months | | | | |

Supplementary Table 4. Summary of Cox proportional hazard regression of *ALG5* and clinical prognostic factors in TTC, HH and UVA-55 cohorts.

|  |  | **Univariate** | | | **Multivariable†** | | | |
| --- | --- | --- | --- | --- | --- | --- | --- | --- |
| Cohort | Gene symbol | Hazard ratio | 95% CI | p-value | Hazard ratio | 95% CI | p-value |  |
| **TTC** | ALG5 (218203_at) | 2.42 | 1.57-3.75 | **p< 0.0001** | 1.88 | 1.11-3.19 | **0.0185** |  |
|  | Stage (III vs IV) | 1.19 | 0.545-2.58 | 0.668 | 1.51 | 0.624-3.66 | 0.361 |  |
|  | Age (≤60 vs >60) | 1.13 | 0.658-1.94 | 0.659 | 1.08 | 0.630-1.86 | 0.773 |  |
| **HH** | ALG5 | 1.60 | 1.03-2.49 | **0.0368** | 1.62 | 1.06-2.47 | **0.0255** |  |
|  | Stage (III vs IV) | 1.78 | 0.796-3.99 | 0.16 | 3.08 | 1.15-8.24 | **0.0248** |  |
|  | Age (≤60 vs >60) | 0.659 | 0.312-1.39 | 0.275 | 0.371 | 0.148-0.926 | **0.0336** |  |
| **UVA-55** | ALG5 (218203_at) | 3.08 | 1.07-8.81 | **0.0365** | 3.08 | 1.07-8.81 | **0.0365** |  |
|  | Stage (III vs IV)* | NA | NA | NA | NA | NA | NA |  |
|  | Age (≤60 vs >60)* | NA | NA | NA | NA | NA | NA |  |
| †Multivariable analysis adjusted for stage and age.  *Not enough cases in stage IV or >60 categories | | | | | | | | |
